# Development and Biotechnological Application of a Novel Endoxylanase Family GH10 Identified from Sugarcane Soil Metagenome

**DOI:** 10.1371/journal.pone.0070014

**Published:** 2013-07-29

**Authors:** Thabata M. Alvarez, Rosana Goldbeck, Camila Ramos dos Santos, Douglas A. A. Paixão, Thiago A. Gonçalves, João Paulo L. Franco Cairo, Rodrigo Ferreira Almeida, Isabela de Oliveira Pereira, George Jackson, Junio Cota, Fernanda Büchli, Ana Paula Citadini, Roberto Ruller, Carla Cristina Polo, Mario de Oliveira Neto, Mário T. Murakami, Fabio M. Squina

**Affiliations:** 1 Laboratório Nacional de Ciência e Tecnologia do Bioetanol (CTBE), Centro Nacional de Pesquisa em Energia e Materiais (CNPEM), Campinas, São Paulo, Brasil; 2 Departamento de Bioquímica, Instituto de Biologia (IB), Universidade Estadual de Campinas (UNICAMP), Campinas, São Paulo, Brasil; 3 Laboratório Nacional de Biociências (LNBio), Centro Nacional de Pesquisa em Energia e Materiais (CNPEM), Campinas, São Paulo, Brasil; 4 Departamento de Física e Biofísica, Instituto de Biociências, Universidade Estadual Paulista (UNESP), Botucatu, São Paulo, Brasil; Auburn University, United States of America

## Abstract

Metagenomics has been widely employed for discovery of new enzymes and pathways to conversion of lignocellulosic biomass to fuels and chemicals. In this context, the present study reports the isolation, recombinant expression, biochemical and structural characterization of a novel endoxylanase family GH10 (SCXyl) identified from sugarcane soil metagenome. The recombinant SCXyl was highly active against xylan from beechwood and showed optimal enzyme activity at pH 6,0 and 45°C. The crystal structure was solved at 2.75 Å resolution, revealing the classical (β/α)_8_-barrel fold with a conserved active-site pocket and an inherent flexibility of the Trp281-Arg291 loop that can adopt distinct conformational states depending on substrate binding. The capillary electrophoresis analysis of degradation products evidenced that the enzyme displays unusual capacity to degrade small xylooligosaccharides, such as xylotriose, which is consistent to the hydrophobic contacts at the +1 subsite and low-binding energies of subsites that are distant from the site of hydrolysis. The main reaction products from xylan polymers and phosphoric acid-pretreated sugarcane bagasse (PASB) were xylooligosaccharides, but, after a longer incubation time, xylobiose and xylose were also formed. Moreover, the use of SCXyl as pre-treatment step of PASB, prior to the addition of commercial cellulolytic cocktail, significantly enhanced the saccharification process. All these characteristics demonstrate the advantageous application of this enzyme in several biotechnological processes in food and feed industry and also in the enzymatic pretreatment of biomass for feedstock and ethanol production.

## Introduction

The conversion of lignocellulosic biomass into renewable fuels has been considered a promising technology to replace fossil fuels and to attend the global need for clean energy. Although the recent worldwide growth in the production of biofuels from plant biomass, several technological bottlenecks still exist and this bioconversion process is not profitable yet [1]. The development of low cost biocatalysts and the improvement of the catalytic efficiency are considered the key to the future of biofuels [1], [2], [3], [4].

Xylanases (E.C. 3.2.1.8) are hemicellulases responsible for breaking down xylan, the major hemicellulosic component of plant cell walls, into short xylooligosaccharides by a general acid–base mechanism involving two glutamic acid residues [5], [6]. Typically, these enzymes can be classified into glycoside hydrolase (GH) families 10 and 11 based on amino-acid sequence similarities [7]. Recently, these enzymes have received much attention owing to their use in degradation of lignocellulosic biomass for biofuels production [8], [9].

The development of novel enzymes is primarily dependent on the effectiveness of the screening strategy and the variety of candidate microorganisms present in certain environmental location [10]. In addition, less than 1% of microorganisms from natural environments can be cultivated using traditional culture methods [11]. Thus, researchers have developed strategies to prospect genes through culture-independent methods [1]. Metagenomics is an advanced strategy to seek for novel functional genes and/or biologically active compounds, by means of direct extraction of all microbial genomic DNA from an environmental sample [12], [13].

Novel xylanases with designed characteristics for biotechnological application have been identified by metagenomics approaches [14]–[16]. A novel GH11 was retrieved from a compost-soil metagenome with thermo-alkali-stability properties, which are of great interest for paper and pulp industry [17]. The GH10 xylanase retrieved from a soil-derived metagenomic library, which unlike most of the xylanases, did not show significantly enzymatic activity inhibition in the presence of metal ions such as Cu^2+^, Zn^2+^ and Co^2+^ [18].

The present study reports the development of a novel endoxylanase family GH10 derived from sugarcane soil metagenome (SCXyl). Along with a comprehensive functional and biophysical characterization, three dimensional structure resolution and SAXS studies of the protein in solution, we also described the potential biotechnological application of this enzyme for biomass to bioproducts application. To the best of our knowledge, this is the first report of a three-dimensional structure for a GH10 derived from a metagenomic library. Collectively, our findings bring relevant insights on enzymatic mechanisms for production of added-value products from plant biomass.

## Materials and Methods

### Ethics Statement

No specific permits were required for the described field studies. The sample of soil used in this work did not involve endangered or protected species and the land field owner approved the sample collection.

### Screening for Xylanase Activity

A sample of soil was colleted in the city of São Carlos (SP), Brazil, at a sugarcane land field after the plantation was harvested. Once the soil was covered with straws, it was expected an enrichment of the microbial population involved in lignocellulose degradation at this location. The metagenomic DNA was extracted from the soil sample (using the FastDNA® SPIN Kit for Soil and the FastPrep® Instrument; MP Biomedicals, Santa Ana,CA) and partially digested with *Sau*3AI. The DNA fragments ranging from 2 to 5 kb were recovered from agarose gel (1.0%, w/v), purified using illustra GFX PCR DNA and Gel Band Purification Kit (GE Healthcare, UK), and then cloned into *Bam*HI digested and dephosphorylated pUC 19 vector (Fermentas- Thermo Scientific, USA). To perform the functional screening, the clones of the metagenomic library were spotted on LB agar plates and incubated overnight at 37°C. Then, the plates were overlaid with agar containing 0.5% (w/v) xylan beechwood (Sigma- Aldrich, USA), incubated for 5 hours at 50°C followed by staining with Congo red, which enabled the visualization of a yellow halo around the positive clone [19].

### Sequence Analyses of the *scxyl* Gene

After functional screening, one positive clone harbouring a 7 kb insert was identified. The plasmid was extracted and submitted to *Sau*3AI restriction assay, producing fragments ranging from 1 to 2 kb that were used for generation of a new DNA library. After cloning the fragments into pUC 19 vector, another round of functional screening was performed. This strategy allowed the easily mapping of the *scxyl* gene responsible for the xylanase activity by DNA sequencing, using M13 forward and reverse primers. The sequencing was performed with the BigDye kit on an ABI Prism 377 Genetic Analyzer (Applied Biosystems, USA) at the Brazilian Bioethanol Science and Technology Laboratory. De-novo assembly of sequence reads and identification of ORFs were performed using Geneious Pro 4.8.5. The ORFs were then analyzed by BLASTx tool from NCBI website. The *scxyl* gene nucleotide sequence was deposited in GenBank database (accession number KC904514). Physical and chemical parameters were predicted using the ProtParam tool from ExPASy (http://web.expasy.org/protparam/). The sequence of amino-acid residues from *scxyl* gene was aligned with reference sequences from non-redundant NCBI database using the ClustalX 1.83 program [20]. The phylogenetic tree was constructed using the Mega 4 program [21] using the neighbor-joining method [22].

### Cloning, Expression and Purification

The coding DNA sequence for SCXyl was PCR-amplified using the forward and reverse primers 5′TATATATCATATGTCTATTTCACGTCGATTATTCC3′ and 5′ATAGGATCCTTACTTCTTCAAATCCAGC3′, respectively. The amplified sequence was cloned into pET28a (Novagen), using *Nde*I and *Bam*HI restriction sites, which carries a fusion sequence encoding a 6×His tag at the N-terminus of the expressed protein.

After confirmation by sequencing, the expression plasmid pET28a (+)-SCXyl was transformed into *E. coli* Rosetta (DE3) competent cells for protein expression. The expression cells were grown in LB broth containing 50 µg ml**^−^** ^1^ kanamycin at 37°C until the OD_600_ reached 0.5–0.6. Afterward, IPTG was added at final concentration of 0.5 mM to induce the expression and the temperature was reduced to 30°C. After 4 h of induction, the cells were harvested by centrifugation and resuspended in lysis buffer (50 mM Tris–HCl pH 7.4, 100 mM NaCl and 5 mM imidazol). The suspension was sonicated after treatment with 0.5 mg/ml lysozyme and 50 µg/ml DNase I. The solution was centrifuged at 10,000 g for 30 min and the supernatant was loaded onto a 5 mL HiTrap™ Chelating HP column (GE Healthcare). The chromatography was carried out using a non-linear imidazole gradient from 5 to 0.5 M with 20 column volumes. To attain a homogenous sample, the sample was further loaded on a gel filtration Superdex 75 10/300 GL column (GE Healthcare), which was previous equilibrated with a 20 mM phosphate buffer (pH 7.4) with 50 mM NaCl. The purified endoxylanase SCXyl was further analyzed by SDS–PAGE under reducing conditions. The protein concentration was assessed by absorbance at 280 nm.

### Enzymatic Assays

The enzymatic assays for endoxylanase SCXyl were performed following Squina et al. [23], where 50 µl of substrate solution (0.5% polysaccharide content) in 100 mM sodium acetate buffer (pH 6.0) was incubated with 10 µl of diluted enzyme in a defined temperature. The enzymatic activity was determined from the amount of reducing sugar liberated from different polysaccharide substrates (purchased from Megazyme, Ireland and Sigma–Aldrich, USA), following the DNS method [24]. One unit of enzyme was defined as the quantity of enzyme that released reducing sugar at a rate of 1 µmol/min. The substrate specificity was evaluated against a set of natural polysaccharides at 50°C using 100 mM sodium acetate buffer pH 6.0 during 10 min. Metal ions and other small compounds were added to the enzyme assay to evaluate the effect on activity [23]. To determine the optimum pH and temperature profiles was applied central composite rotatable design (CCRD), where the enzymatic reaction was carried out at different pHs (3.0, 3.7, 5.5, 7.3, 8.0) in 100 mM phosphate citrate buffer and at a range of temperatures (20, 27, 35, 63, 70°C). For thermostability evaluation, enzyme was incubated at 40, 50, 60, 70, 80°C, for 30 s to 6 h. After, an aliquot of enzyme was taken and the residual activity was measured.

The apparent kinetics parameters K_m_, V_max_, k_cat_, and k_cat_/K_m_ were calculated from initial velocities at substrate concentration of xylan varying from 0.625 to 11.25 mg/mL. Assays were conducted in 100 mM sodium acetate buffer (pH 6.0) at 45°C for 5 min. Mathematical calculations were made using the software Graph Pad Prism 5.0 (GraphPad Software). These assays were performed in quintuplicate.

Evaluation of biomass conversion was performed using phosphoric acid-pretreated (2.5% *w/v*) sugarcane bagasse (PASB), whose composition was determined to be 48.5% of cellulose, 17.0% of hemicellulose, 28.8% of lignin and 3.8% of ashes. In this case, the reaction containing 1.0% of substrate and 100 mM sodium acetate buffer at pH 6.0 were incubated with 10 µg of SCXyl at 40°C during 24 h under constant agitation. After the incubation period, the impact of SCXyl to enhance the cellulose fibers digestibility were evaluated by the addition of 1.4 µg of ACCELLERASE® 1500 (Genencor, Netherlands) and the incubation at 40°C for 23.5 h. Then, the supernatant was separated from residual polysaccharides and analyzed through the DNS method.

### Analysis of Hydrolysis Products

We studied the mode of operation of endoxylanase SCXyl by incubating it with 0.1 M of xylobiose (X2), xylotriose (X3), xylotetraose (X4), xylopentaose (X5) and xylohexaose (X6) at 45°C for 30 min and for 16 h. The hydrolysis product from 0.5% beechwood, 0.5% wheat arabinoxylan or 1% PASB were also analyzed after 16 h of incubation period, The products were derivatized with 8-aminopyrene-1,3,6-trisulfonic acid (APTS) by reductive amination as described previously [25]. Capillary electrophoresis of oligosaccharides was performed using a P/ACE MDQ system (Beckman Coulter) with laser-induced fluorescence detection. A fused-silica capillary (TSP050375, Polymicro Technologies) of internal diameter 50 µm and length of 31 cm was used as the separation column for the oligosaccharides. Samples were injected by application of 0.5 psi for 0.5 s. Electrophoresis conditions were 15 kV/70–100 µA with the cathode at the inlet, 0.1 M sodium phosphate pH 2.5 as running buffer and a controlled temperature of 20°C. The capillary was rinsed with 1 M NaOH followed by running buffer with a dip-cycle to prevent carry over after injection. Oligomers labeled with APTS were excited at 488 nm and emission was collected through a 520 nm band pass filter. Because of the small volumes of capillary electrophoresis combined with small variations in buffer strength, retention times varied slightly when comparing separate electrophoresis runs. The combined information obtained from the electrophoretic behavior and co-electrophoresis with mono and oligosaccharides standards (purchased from Sigma and Megazyme) was used to identify the degradation products.

### Spectroscopic Methods

Far-UV circular dichroism (CD) measurements (195–250 nm) were carried out using a JASCO 815 spectropolarimeter (JASCO Inc., Tokyo, Japan) equipped with a Peltier temperature control unit using a 0.1 cm path quartz cuvette. The solvent spectra were subtracted in all experiments to eliminate background effects. The CD spectra were the average of 8 accumulations using a scanning speed of 100 nm min**^−^** ^1^, spectral bandwidth of 1 nm, and response time of 0.5 s. The protein concentration was 0.2 mg/mL in 50 mM sodium phosphate buffer at pH 7.4. The thermal denaturation of the enzyme was characterized by measuring the ellipticity changes at 222.6 nm induced by a temperature increase from 20 to 100°C at a heating rate of 1°C min**^−^** ^1^ [25].

### Small Angle X-ray Scattering

Small angle X-ray scattering (SAXS) data were collected on the SAXS2 beamline at the Brazilian Synchrotron Light Laboratory. The radiation wavelength was set to 1.48 Å and a 165 mm MarCCD detector was used to record the scattering patterns. The sample-detector distance was set to 1084.42 mm to give a scattering vector range from 0.016 to 0.32 Å^−1^. Protein samples were prepared in 20 mM phosphate buffer (pH 6.0) at 5 and 1 mg ml^−1^. Fitting of the experimental data and evaluation of the pair–distance distribution function *p*(r) were performed using the program GNOM [26]. Molecular weight was evaluated from SAXS curve using SAXSmoW [27]. The low-resolution dummy residues (DR) model of SCXyl was determined using *ab initio* modeling as implemented in the program GASBOR [28]. Ten different models were generated and the best model was choosed based on normalized spatial discrepancy using the suite of programs DAMAVER [29]. CRYSOL [27] was used to evaluate simulated SAXS curve and structural parameters from crystallographic structure. The DR model and the crystallographic structure were superimposed using the program SUPCOMB [29].

### Crystallization, Data Collection and Processing

SCXyl was dialyzed against 20 mM Tris-HCl buffer (pH 7.5) and concentrated to 7.4 mg ml^−1^ using Amicon Ultra-4 centrifugal filter units (Millipore). Crystallization screening was performed by vapor diffusion method. Sitting drops were prepared using a HoneyBee 963 robot (Genomic Solutions) by mixing 0.5 µl of protein solution with an equal volume of the mother liquor and equilibrated against 80 µl of reservoir at 18°C. Based on commercially available kits (SaltRX, Crystal Screen and Crystal Screen 2– Hampton Research, Precipitant Synergy, Wizard I and II – Emerald BioSystems, PACT and JCSG – Qiagen/Nextal) five hundred forty four solutions were tested. Automated imaging of crystallization plates was carried out using the Rock Imager Robot (Formulatrix). Clusters of needles were obtained in one week in the drop containing 20% (*w/v*) PEG 8000, 200 mM sodium chloride and 100 mM sodium phosphate/citrate buffer, pH 4.2 (Figure S1A). The crystal optimization consisted of varying pH, precipitant concentration and additives, by both sitting and hanging drop methods. Three-dimensional crystals (Figure S1B) were obtained in three weeks in hanging drops containing 5% (*v/v*) glycerol, 20% (*w/v*) PEG 8000, 200 mM sodium chloride and 100 mM sodium phosphate/citrate buffer, pH 4.2. One crystal was directly flash cooled in nitrogen gas stream at 100 K since the mother liquor was already cryoprotectant. X-ray diffraction data were collected at the W01B-MX2 beamline (Brazilian Synchrotron Light Laboratory, Campinas, Brazil). Data were indexed, integrated and scaled using the HKL2000 package [30]. Calculations based on the molecular weight of 40 kDa for one monomer, indicated the presence of two protomers in the asymmetric unit, with a solvent content of 50% and a Matthews coefficient of 2.49 Å^3^ Da^−1^.

### Structure Determination and Refinement

The structure was solved by molecular-replacement (MR) method using the atomic coordinates of CmXyn10B (PDB code: 2CNC) as the search model in the Phaser software from PHENIX package [31]. Model building was initially performed using the AutoBuild Wizard from PHENIX [31], which employs several cycles of automated protein chain tracing, iterative density modification and restrained refinement. Further refinements steps involved manual inspection and rebuilding of protein chains using COOT [32] intersperse with restrained refinement with phenix.refine routine from PHENIX [31] using 7 TLS groups: 32∶106, 107∶135, 136∶275, 276∶379, 34∶263, 264∶306 and 307∶378. The final coordinates comprise of residues Arg^32^-Lys^379^ (monomer A) and Gly^34^-Lys^378^ (monomer B). The Trp^281^-Arg^291^ loop was disordered in chain A and it could not be modeled. Global and local stereochemistry of the final structure was verified using the Molprobity server [33].

### Protein Data Bank Accession Code

The atomic coordinates and structure factors of SCXyl have been deposited with the Protein Data Bank under the accession code 4K68.

## Results and Discussion

### Phylogenetic Analysis of SCXyl

A metagenomic library with approximately 26.900 clones was constructed with the DNA extracted from a sugarcane fiel soil. The restriction analysis evidenced that the insert size of the metagenomic library ranged from 1 to 8 kb with an average size of 3.5 kb. After screening the metagenomic library using xylan beechwood as substrate, it was possible to identify one positive clone displaying xylanase activity. The restriction analysis of the positive plasmid showed that the size the insert was about 7 kb. After subcloning this DNA fragment, it was possible to identify one clone harboring a 2 kb DNA fragment displaying xylanase activity. Sequence analysis fragment revealed an ORF encoding a protein with 380 aminoacid residues containing a 27-residue long putative signal peptide. The amino acid sequence of SCXyl showed the highest identity (61%) with the xylanase Xyn10b from *Cellvibrio mixtus,* (CmXyn10b, GenBank accession numberAAD09439), followed by a xylanase from *Cellvibrio japonicus* Ueda 107 (60% of identity, GenBank accession number ACE84280) and a xylanase from uncultured bacterium (59% of identity, GenBank accession number ADK78237). The molecular weight and isoeletric point calculated for mature protein SCXyl were 40.6 kDa and 6.7, respectively. The apparent molecular weight based on SDS-PAGE was about 40 kDa (Figure S2).

**Figure 1:**
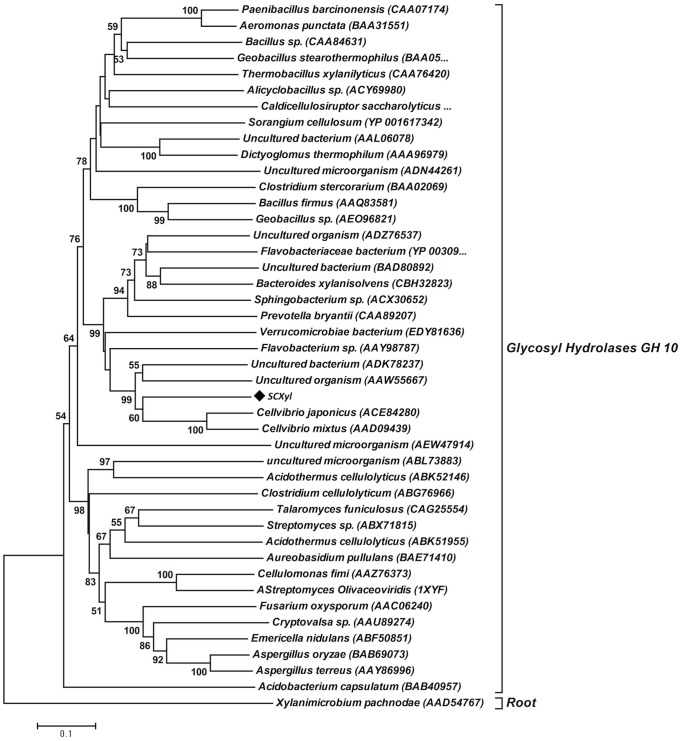
Phylogenetic relationships among members of Glycoside Hydrolase Family 10. The phylogenetic tree was generated using the amino acid sequences from bacterial, fungal and uncultured microorganisms GH10 family members and one bacterial GH11 member (root). This tree was constructed using MEGA 4.0 software by the Neighbour Joining (NJ) method. The Bootstrap values (n = 1000 replicates) are indicated as percentage at the node of phylogenetic tree.

The phylogenetic tree evidenced the closest relationship of SCXyl with xylanases from *Cellvibrio spp.* and uncultured microrganisms, which together were grouped at the same clade, where SCXyl can be considered unaffiliated GH10 member within this specific clade (Figure 1). The multi-alignment analysis evidenced that ScXyl is very diverse GH10 from previously characterized xylanases (data not shown).

### Biochemical Characterization

The mature protein without the signal peptide, containing a 6×His-Tag at the N-terminus, was successfully overexpressed in the cytoplasmic fraction of *E. coli Rosetta (DE3),* after induction by IPTG for 4 h at 30°C. The protein purification steps included Ni^2+^-chelating affinity and size exclusion chromatography and resulted in a highly purified sample suitable for biochemical and biophysical assays (Figure S2).

**Figure 2:**
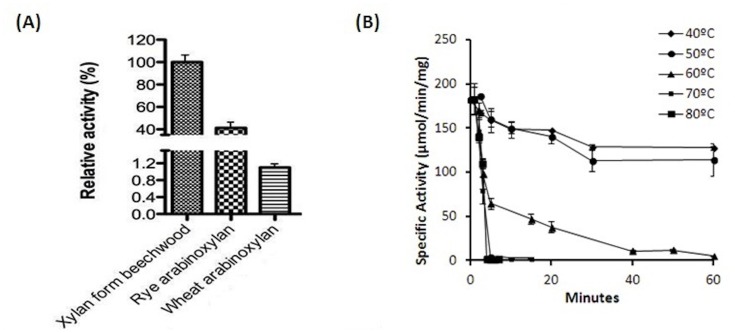
Biochemical characterization of SCXyl. (A) The substrate specificity of endoxylanase SCXyl against three types of xylan and (B) the thermal stability in different temperatures of incubation are shown.

The substrate specificity analysis revealed that the recombinant endoxylanase SCXyl can efficiently digest beechwood xylan (Figure 2A). Beechwood xylan is 95% soluble in water and is composed of a high percentage of neutral sugars (∼ 97% mainly xylose residues; small amounts of glucose, and traces of arabinose and galactose can be found) and less than 3% hexuronic acids [34]. The endoxylanase SCXyl also was able to degrade rye arabinoxylan (the relative activity compared to beechwood xylan was 41.3%) and wheat arabinoxylan (1.1%) as can be visualized in the Figure 2A. Other substrates were also parsed, including debranch arabinan, xyloglucan, β-glucan, galactoglucan, and carboxymethylcellulase, where no activity was evidenced, as expected for a GH10 member. In relation to thermal stability, SCXyl lost its activity after 15 min of incubation at 80 and 70°C and 60 min at 60°C (Figure 2B). The enzyme was stable at 50 and 40°C retaining more than 60% its initial activity after 6 h of incubation (data not shown). The effect of ions, NaCl, EDTA and EGTA on the catalytic activity of endoxylanase SCXyl was also evaluated. The results presented in Table 1 reports that SCXyl was highly inhibited by Co^2+^, Zn^2+^, Mn^2+^, Cu^2+^, Ni^2+^ and Fe^3+^ (at 5 mM). The other salts evaluated showed low effect on the enzymatic activity.

**Table 1:** Effect of ions, EDTA and EGTA on the catalytic activity of endoxylanase SCXy1 from sugarcane soil metagenome.

Ions or ChemicalReagents	Concentration (mM)	Relative Activity (%)
Control	–	100.00
EDTA	10 mM	92.01±1.92
EGTA	10 mM	85.08±2.55
CaCl_2_	5 mM	93.56±9.82
ZnSO_4_	5 mM	0.00
MnCl_2_	5 mM	50.19±6.25
MgCl_2_	5 mM	89.24±7.82
CuSO_4_	5 mM	0.00
LiCl	5 mM	99.04±1.86
KCl	5 mM	107.06±7.08
NaCl	5 mM	95.98±3.99
CoCl_2_	5 mM	48.12±12.9
FeCl_3_	5 mM	4.28±0.27
NiCl_2_	5 mM	29.29±5.73

**Figure 3:**
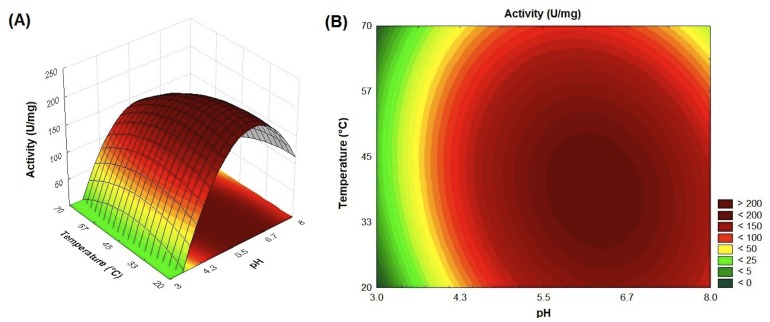
Temperature and pH profile of SCXyl. (A) The surface response and (B) contour curves generated in the central composite rotatable design (CCRD) illustrate the effect of the pH and temperature on the xylanase activity.

The effects of pH and temperature on the SCXyl catalytic activity was evaluated (Figure S3). The purified xylanase showed optimum activity at 45°C although it retained more than 60% from 20°C to 50°C. The optimal pH of the recombinant enzyme was 6, but the activity was noticed over a broad pH range, from 4 to 9 (Figure S3). A central composite rotatable design (CCRD) using pH and temperatures as the variables was performed (Table S1). According to the analysis of variance (ANOVA), the model was significant at high confidence level (95%), with R^2^ = 0.95. The best condition for enzyme activity was reached at pH 6.0 and temperature 45°C (Figures 3A and 3B). The optimum temperature and pH reported for endoxylanase SCXyl is comparable to other GH10 endoxylanases described in literature. For xylanases from *Plectophaerella cucumerina* (XynZC) [35], *Clostridium cellulovorans* (XynB) [36] and *Thichoderma harzianum T4* [37], the optimum temperature was 40°C. For the xylanase from alpine tundra soil [38], the optimum pH range was 6.0–6.5.

The apparent kinetic values were determined under optimal pH and temperature from the initial rates of beechwood xylan hydrolysis in various substrate concentration. The calculated K_m_ and V_max_ were 2.18±0.13 mg/ml and 1,43±30.42 µmol/min.mg, respectively. The values for k_cat_ (catalytic constant) and k_cat/_K_m_ (catalytic efficiency) were 1.8 s^−1^ and 496.3 ml/mg.s^−1^, respectively. The xylanases (GH10) from *Thermotoga thermarum* [39] and *Glaciecola mesophila* [40], also evaluated using xylan beechwood, exhibited a k_cat/_K_m_ of 276.37 ml/mg.s^−1^ and 56.56 ml/mg.s^−1^, respectively. Thus, sugesting that SCXyl present a higher catalytic efficiency in comparison to other known xylanases.

**Figure 4:**
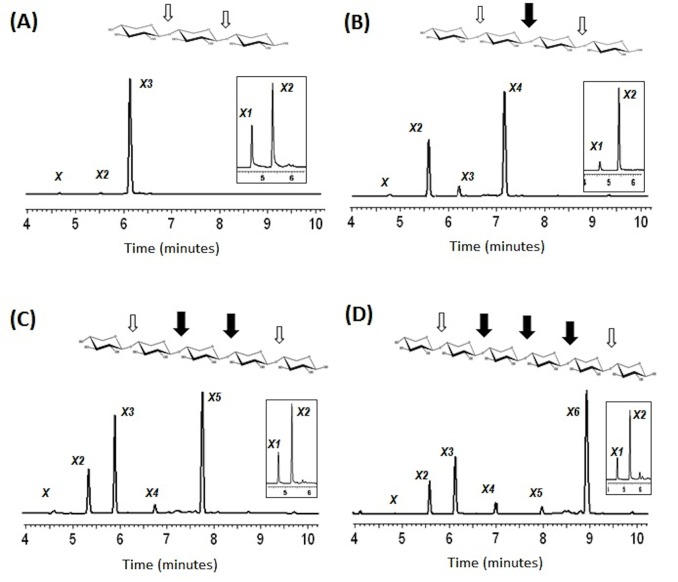
Analysis of the breakdown products released by SCXyl. Xylotriose, xylotetraose, xylopentaose and xylohexaose degradation profiles are represented, respectively, (A), (B), (C) and (D). The intermediary products after 30 min of incubation are represented. The detached small boxes show the final degradation products, which were always xylose and xylobiose. The black and white arrows depict the preferential and the less preferential SCXyl cleavage site, respectively, based on the profile of intermediary products formed.

The analysis of oligosaccharides degradation through capillary electrophoresis (Figure 4) showed that SCXyl was able to degrade all the oligosaccharides tested with the exception of xylobiose (data not shown). It was possible to observe the production of intermediary xylo-oligosaccharides after short time incubations. The X3 was poorly hydrolyzed after 30 min of reaction (Figure 4A). As a result of continued incubation, SCXyl completely hydrolyzed X3, X4, X5 and X6 to produce only xylose and xylobiose (Figure 4A–D). According to our data, oligosaccharide breakdown occurred preferentially in the internal glycosidic bonds, clearly seen after the X4 hydrolysis and the main formation of X2 (Figure 4B).

### Biophysical Characterization

**Figure 5:**
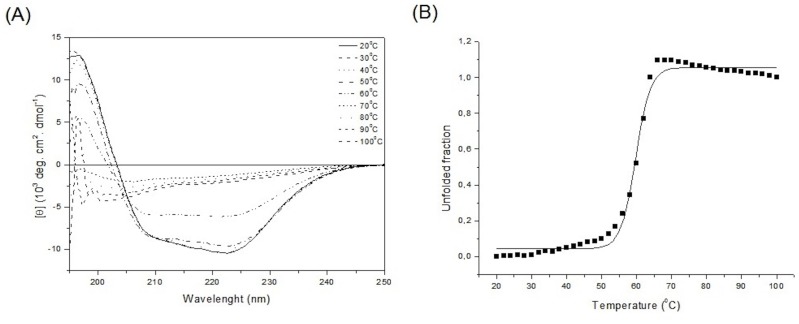
Biophysical characterization of SCXy11. *(*A) Far UV-CD spectrum of SCXyl at 20°C and (B) Thermal stability of SCXyl by CD.

Far-UV CD spectrum of SCXyl presented two minimal points at 222 nm and 209 nm, and a maximum at 197 nm (Figure 5A) as expected for GH10 members exhibiting a TIM-barrel fold. The minimum at 222 nm is related to the presence of α-helical elements and the other at 209 nm has a contribution of both α and β secondary structure elements. All these data together indicates that the secondary structure of SCXyl is composed by α-helical and β-structures (as observed in the crystal structure) and indicated a well-folded protein. We also investigated the thermal stability of SCXyl by CD spectroscopy, and it resulted in a melting temperature (Tm) of 59.7°C (Figure 5B), which is consistent with the previous result about the rapid loss of enzyme activity in temperatures above 60°C.

### Overall Structure of SCXyl

**Figure 6:**
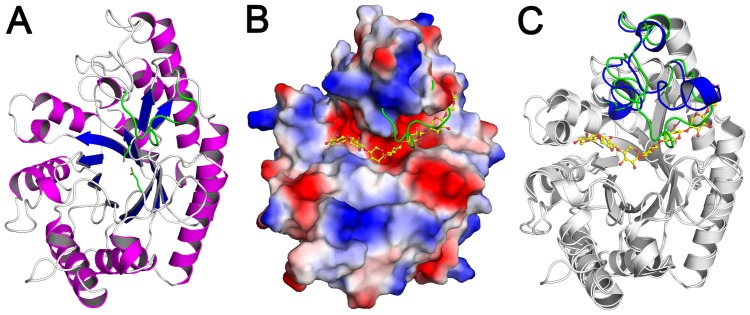
The three-dimensional structure of SCXyl. A) Cartoon representation, colored by secondary structure, with the Trp281-Arg291 loop (chain B) colored in green and the catalytic residues shown as sticks. B) Surface electrostatic potential, colored from negative (red) to positive (blue) charge. The ordered loop in chain B is shown as a green line. C) Superposition of CmXyn10B (PDB code 1UQY) on SCXyl structure, with divergent regions in blue and green, respectively. The substrate from the CmXyn10B-complex structure is represented as ball-and-sticks with carbon atoms in yellow.

The SCXyl enzyme crystallized in the monoclinic space group with two molecules in the asymmetric unit (Table 2). The crystal structure has been determined at 2.75 Å resolution, presenting good overall stereochemistry and crystallographic residuals (Table 2). SCXyl structure comprises the classical (β/α)_8_-barrel fold (commonly referred as TIM barrel) (Figure 6A), with the active site located at the groove formed by the loops connecting the β-strands and the α-helices (Figure 6B). The catalytic residues Glu^166^ (the acid/base) and Glu^271^ (the nucleophile) are located after the fourth and the seventh strands of the barrel, respectively (Figure 6A). The two monomers of SCXyl in the asymmetric unit are very similar, showing r.m.s. deviation of 0.295 Å. The main difference between them is the loop connecting the seventh strand with the next α-helix, which is partially disordered in chain A (Trp^281^-Arg^291^ residues were not modeled) and well-ordered in chain B (Figures 6B and 6C). This loop forms the aglycone region (+ subsites) in the CmXyn10B structure (PDB code 1UQY), exhibiting an open conformation (Figure 6C). In chain B of SCXyl, this loop adopts a closed conformation, being stabilized by contacts with residues considered relevant for substrate recognition and binding such as Tyr^209^, Trp^345^ and Phe^349^ (Figure 6C). The distinct conformational states of this loop in the two SCXyl chains and CmXyn10B structure suggest an inherent flexibility of this region, which becomes ordered in an open conformation upon substrate binding. Interestingly, this loop is remarkably shortened (only three residues long) in the hyperthermophilic xylanase 10B from *Thermotoga petrophila* RKU-1 [4], indicating a possible correlation of this loop with thermostability.

**Table 2:** Data collection and refinement statistics for the GH10 endoxylanase (SCXy1) retrieved from sugarcane soil metagenome.

	SCXy1
***Data collection and processing***	
X-ray Source	MX2 beamline (LNLS, Campinas, Brazil)
Wavelength (Å)	1.459
Temperature (K)	100
Space group	P2_1_
Cell dimensions (Å,°)	a = 41.88, b = 116.44, c = 82.81; β = 99.85
Resolution (Å)	47.40-2.75
No. of unique reflections	20,135
R_merge_ (%)	14.3 (46.8)
<I/σ(I)>	9.18 (2.38)
Completeness (%)	97.9 (87.2)
Multiplicity	4.4 (3.8)
Number of molecules in the asymmetric unit	2
Solvent Content (%)	48.5
V_M_ (Å^3^.Da^−1^)	2.39
***Refinement***	
R_factor_ (%)	20.36 (28.18)
R_free_ (%)	25.52 (31.85)
r.m.s. deviation bond distances (Å)	0.002
r.m.s. deviation bond angles (°)	0.590
Average B-factor (Å^2^)	47.30
Ramachandran analysis	
Favored (%)	97.2
Allowed (%)	2.5
Outliers (%)	0.3

Statistical values for the highest-resolution shells are given in parentheses.

### Structural Mapping of Substrate-binding Sites

**Figure 7:**
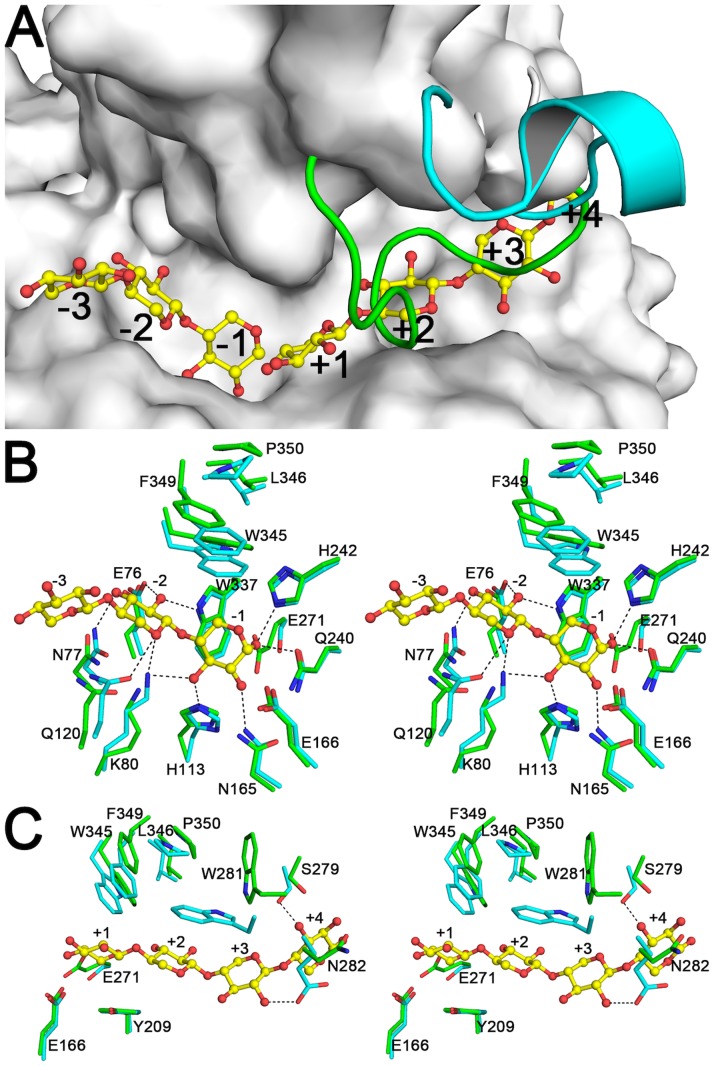
Substrate binding sites of SCXyl. A) Molecular surface of chain A, with the Trp281-Arg291 loop from chain B and CmXyn10B shown as green and blue lines, respectively. B) Stereo view of the glycone region (from −3 to −1 subsites) of SCXyl (carbon atoms in green) and CmXyn10B (carbon atoms in blue). C) Stereo view of the aglycone region (from +1 to +4 subsites). The substrate found in the CmXyn10B-complex structure (PDB code: 1UQY) is shown in Figs. A, B and C, as ball-and-sticks. Residues numbering refers to the SCXyl enzyme.

The structural superposition of the CmXyn10B in complex with xylopentaose [41] on the SCXyl structure revealed a very conserved active-site pocket, including all residues involved in the substrate recognition and binding (Figure 7A). By analogy, the residues Glu^76^, Lys^80^, Gln^120^ and Trp^337^ comprise the −2 subsite and are involved in the interaction with oxygen atoms from xylosyl groups (Figure 7B). These interactions have been shown to be crucial for catalytic activity of GH10 xylanases, since mutation of any of these residues to alanine reduces enzymatic activity against xylooligosaccharides [42]. The −1 subsite is formed by the aromatic Trp^345^ and polar residues His^113^, Asn^165^, Gln^240^ and His^242^ (Figure 7B). Trp^345^ plays an essential role orientating the xylosyl moiety at the −1 position to permit the action of the nucleophile (Glu271) on the glycosidic scissile bond. As in CmXyn10B, the Trp^345^ mobility is restricted by Phe^349^, Leu^346^ and Pro^350^ residues (Figure 7B) resulting in a stable side-chain rotamer conformation. Hydrophobic interactions at the +1 subsite have been proposed to be determinant for the high activity of CmXyn10B against small xylooligosaccharides [41]. In the SCXyl structure, all these residues are fully conserved that could explain the similar preference for xylooligosaccharides as substrate. The xylosyl moiety at the +1 subsite is stabilized by hydrophobic contacts with the aromatic residues Tyr^209^, Trp^345^ and Phe^349^ (Figure 7C). The mutant Phe^340^Ala (corresponding to Phe^349^ in SCXyl) greatly reduced the activity against xylooligosaccharides in CmXyn10B [41]. This observation evidences the importance of this residue to conformational stability of the +1 subsite, as well as in selection of the xylosyl moiety to the −1 subsite.

The Trp^281^-Arg^291^ loop, disordered in the chain A and ordered with a closed conformation in the chain B of the SCXyl structure, is nearly identical to that from CmXyn10B in terms of primary structure (WXLPXAEVSTR). It suggests that this loop in SCXyl should adopt an open conformation to accommodate the substrate at the active-site pocket as observed in the CmXyn10B-substrate complex (Figure 7A). This loop forms the aglycone region; therefore, the +2 and +3 subsites interactions in CmXyn10B may be extrapolated to SCXyl. Trp^281^ should stack against xylosyl moieties at +2 and +3 subsites (Figure 7C). The Glu^273^ of CmXyn10B, which makes hydrogen bond with O2 atom at the +3 subsite (Figure 7C), is substituted in SCXyl by an asparagine residue (Asn^282^), which could preserve the polar contact with the substrate. The Ser^279^ should interact with O3 atom from the xylosyl residue at the +4 subsite (Figure 7C).

The detailed structural mapping of the substrate-binding sites of SCXyl by analogy with CmXyn10B suggests that the high enzymatic activity against small xylooligosaccharides should be related to low-binding energies of subsites that are distant from the site of hydrolysis, as well as by additional hydrophobic contacts at the +1 subsite. These observations are consistent with the previous result (Figure 4) and explain why this enzyme displays unusual capacity to degrade small xylooligosaccharides.

### Low-resolution Molecular Structure

**Figure 8:**
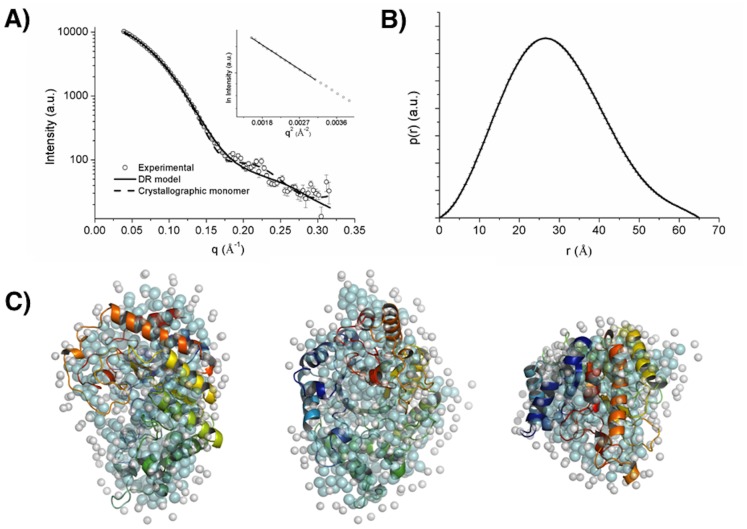
SCXy1 analysis by SAXS. (A) Experimental SAXS curve of the SCXyl and fitting procedures, and experimental distance distribution function. An inset containing the corresponding Guinier plot. (B) DR model (aminoacids are represented by cyan dummy residues and salvation shell by gray dummy residues) superposed with SCXyl crystallographic monomer structure).

To analyze the shape and oligomeric state of endoxylanase SCXyl, we performed SAXS experiment at two concentrations, 1 and 5 mg/mL. Radius of gyration (R_g_) obtained by Guinier plots exhibited similar values, indicating absence of structure factor on data (interparticle correlation). Typical scattering curve and the distance distribution function p(r) obtained with SCXyl preparation at 5 mg ml^−1^ is presented in Figure 8A. The final SAXS envelope (Figure 8A) was chosen based on normalized spatial discrepancy parameter and it fitted well the experimental X-ray scattering curve (χ = 2.82) (Figure 8B). SAXSmoW was used to estimate the oligomeric state of the protein and predicted a molecular weight of 40 kDa indicating that corresponds to a monomer of SCXyl (calculated molecular weight from primary structure: 40.64 kDa). Despite two molecules were found in the asymmetric unit, SAXS data confirmed that SCXyl behaves as a monomer in solution. Thus, the crystallographic monomer was fitted compared to experimental X-ray scattering curve (χ = 4.89) (Figure 8A) indicating a good agreement between the in solution and crystal structures. Structural parameters derived from experimental curve, DR model and crystallographic structure of SCXyl are given in Table 3.

**Table 3:** SAXS structural parameters of endoxylanase SCXy1.

Endoxylanase	Experimental 5/mg/mL	DR model	Crystallographic
**R_g_ (Å) (Guinier)**	23.20	–	–
**R_g_ (Å)**	22.04±0.01	–	21.14
**D_max_ (Å)**	65±5	–	70.90
**Molecular weight (kDa)**	40.00	–	39.84
**Excluded volume (Å)**	50880	–	50250
**SAXS resolution (Å)**	19.63	19.63	–
**X**	–	2.82	4.89

### Biotechnological Application

**Figure 9:**
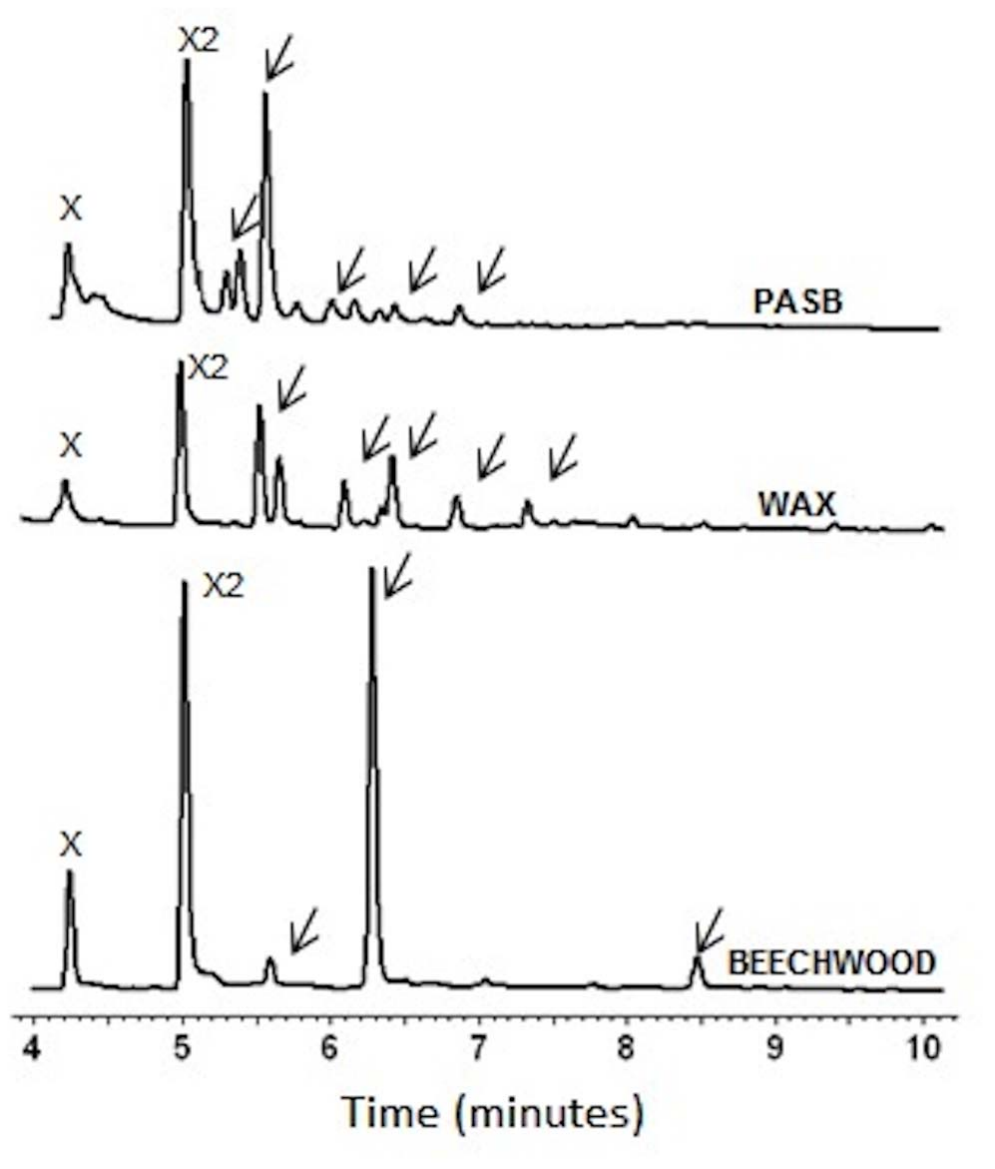
Production of xylo-oligosaccharides and xylose from xylan beechwood, WAX and PASB by SCXyl. Xilose and xilobiose (X and X2, respectively) were produced along with longer oligosaccharides, as indicated by the arrows.

The end products generated after SCXyl hydrolysis of xylan beechwood, wheat arabinoxylan and PASB were analyzed through capillary zone electrophoresis. In all cases, the main products were xylooligosaccharides (XOs), but xylobiose and xylose were also produced (Figure 9). XOs and xylobiose are of great interest for food industry because of its application as prebiotics as well as sweeteners [43]. Moreover, the production of xylose directly from these substrates can be used for the production of xylitol, an alternative sweetener [44].

**Figure 10:**
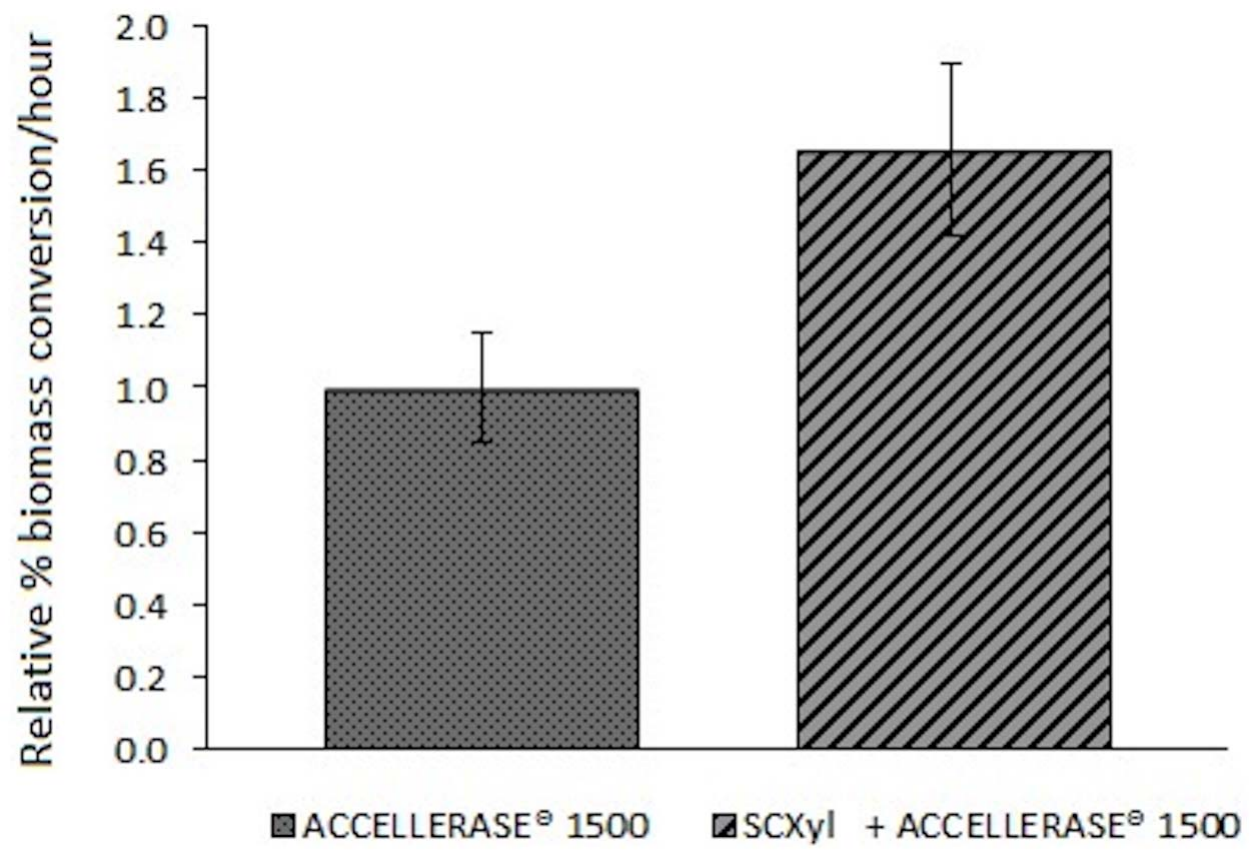
The effect of SCXyl treatment on PASB prior the saccharification with commercial cellulase preparation. The pre-treatment step with SCXyl followed by addition of ACCELLERASE® 1500 (cellulolytic enzymatic cocktail) improved PASB saccharification.

Due to SCXyl ability of realeasing XOs, xylobiose and xylose from PASB we analyzed the effect of the enzymatic treatment prior biomass sacharification by commercial cellulases. It has already been reported that this enzymatic step is advantageous for biomass saccharafication [14]. The SCXyl pre-treatment significantly enhanced the saccharification of PASB by ACCELLERASE® 1500, as the amount of reducing sugars increased approximately 65% (Figure 10). Probably, SCXyl treatment facilitated the access of endo/exoglucanases and β-glucosidases to cellulose microfibrils, which are naturally surrounded by hemicellulose [45]. This result demonstrated the potential application of SCXyl in biofuel production.

### Concluding Remarks

A new xylanase 10B with great biotechnological potential has been isolated from sugarcane soil metagenome and characterized in both functional and structural aspects. The SCXyl has unusual enzymatic activity against small xylooligosaccharides, such as xylotriose, which is result from low-binding energy of distant subsites and from hydrophobic contacts around the site of hydrolysis. This characteristic is advantageous, because this allows the enzyme to maintain active even in advanced steps of the catalysis, when most of the long xylan chains have been cleaved.

SCXyl produces compounds with biotechnological application in food and feed industry. The use of SCXyl as pre-treatment step of PASB, prior to the addition of a commercial cellulolytic cocktail, significantly enhanced the saccharification process. All these characteristics, and the broad range of temperature and pH, turn this enzyme valuable for biotechnological processes. Collectively, our findings shed light on enzymatic mechanisms for xylooligosaccharide production, as well as provide basis for further studies for the development of novel enzymes and enzymatic routes for converting plant biomass into bio-products.

## Supporting Information

Figure S1 **Crystallization of SCXyl.** A) Clusters of needles obtained by sitting-drop vapor-diffusion method in the initial screening. B) Three-dimensional crystals obtained in hanging-drop during optimization steps
(TIF)

Figure S2 **Expression and Purification of SCXyl.** SDS-PAGE analysis of the SCXyl after recombinant expression and chromatographic purification steps.
(TIF)

Figure S3 **The effects of pH and temperature on the SCXyl catalytic activity.** (A) The enzymes was incubated at different pH (pHs 3–9) and (B) temperatures (10–90°C) using beechwood xylan as the substrate.
(TIF)

Table S1 **Matrix of the CCRD (Central Composite Rotational Design) to determine the optimal temperature and pH of endoxylanase SCXyl from sugarcane soil metagenome.**
(DOCX)
