# Supplementary figures and images for: Development and Biotechnological Application of a Novel Endoxylanase Family GH10 Identified from Sugarcane Soil Metagenome
Source: PLoS One. 2013 Jul 29;8(7):e70014. doi: 10.1371/journal.pone.0070014 (PMC3726488; doi:10.1371/journal.pone.0070014)

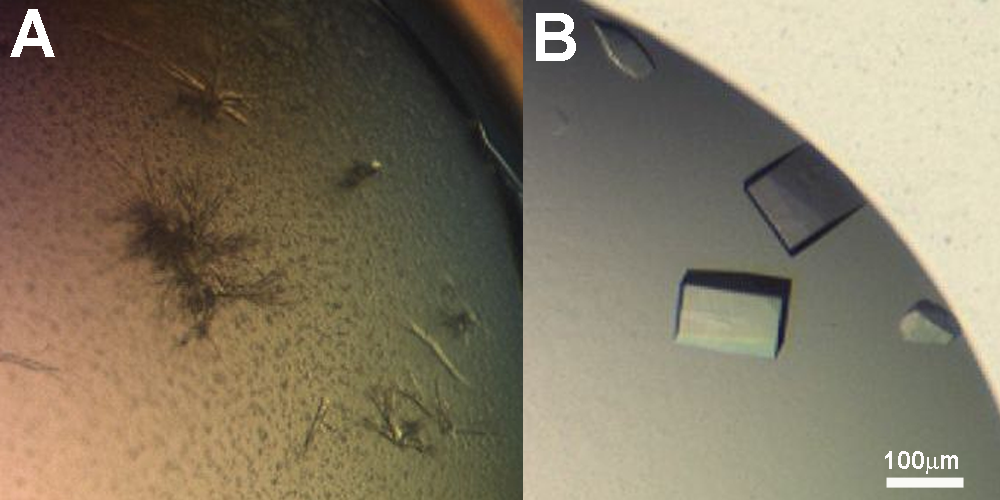

Supplement: Figure S1 — Crystallization of SCXyl. A) Clusters of needles obtained by sitting-drop vapor-diffusion method in the initial screening. B) Three-dimensional crystals obtained in hanging-drop during optimization steps (TIF) [file pone.0070014.s001.tif]

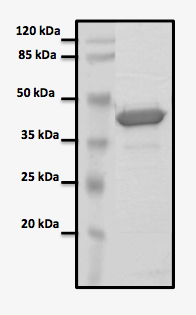

Supplement: Figure S2 — Expression and Purification of SCXyl. SDS-PAGE analysis of the SCXyl after recombinant expression and chromatographic purification steps. (TIF) [file pone.0070014.s002.tif]

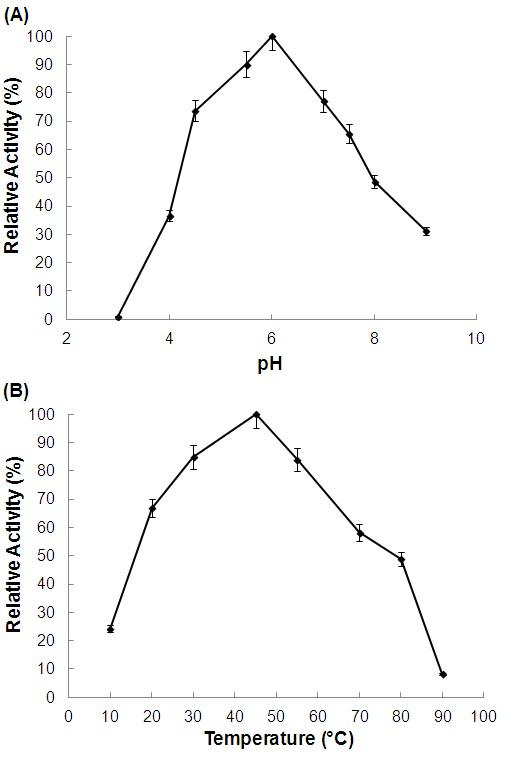

Supplement: Figure S3 — The effects of pH and temperature on the SCXyl catalytic activity. (A) The enzymes was incubated at different pH (pHs 3–9) and (B) temperatures (10–90°C) using beechwood xylan as the substrate. (TIF) [file pone.0070014.s003.tif]
