# Supplementary material for: Development and Biotechnological Application of a Novel Endoxylanase Family GH10 Identified from Sugarcane Soil Metagenome
Source: PLoS One. 2013 Jul 29;8(7):e70014. doi: 10.1371/journal.pone.0070014 (PMC3726488; doi:10.1371/journal.pone.0070014)
Supplement: Table S1 — Matrix of the CCRD (Central Composite Rotational Design) to determine the optimal temperature and pH of endoxylanase SCXyl from sugarcane soil metagenome. (DOCX) [file pone.0070014.s004.docx]

**Table S1.** Matrix of the CCRD (Central Composite Rotational Design) to determine the optimal temperature and pH of endoxylanase SCXyl from sugarcane soil metagenome.

| **Tests** | **pH** | **Temperature (°C)** | **Specific Activity (U/mg)** |
| --- | --- | --- | --- |
| 1 | (-1) 3.7 | (-1) 27 | 3.32 |
| 2 | (+1) 7.3 | (-1) 27 | 177.07 |
| 3 | (-1) 3.7 | (+1) 63 | 2.55 |
| 4 | (+1) 7.3 | (+1) 63 | 116.71 |
| 5 | (-1.41) 3.0 | (0) 45 | 2.08 |
| 6 | (+1.41) 8.0 | (0) 45 | 112.53 |
| 7 | (0) 5.5 | (-1.41) 20 | 160.10 |
| 8 | (0) 5.5 | (+1.41) 70 | 111.05 |
| 9 | (0) 5.5 | (0) 45 | 190.56 |
| 10 | (0) 5.5 | (0) 45 | 184.79 |
| 11 | (0) 5.5 | (0) 45 | 186.55 |
